# Sonographic Correlations With Histological Grade and Biomarker Profiles in Breast Invasive Ductal Carcinoma

**DOI:** 10.1002/cnr2.70288

**Published:** 2025-07-31

**Authors:** Golnaz Moradi, Nasrin Ahmadinejad, Diana Zarei, Nahid Sadighi

**Affiliations:** ^1^ Advanced Diagnostic and Interventional Radiology Research Center (ADIR) Tehran University of Medical Sciences Tehran Iran; ^2^ Department of Radiology, Sina Hospital Tehran University of Medical Sciences Tehran Iran; ^3^ Advanced Diagnostic and Interventional Radiology Research Center (ADIR), Imam Khomeini Hospital, Cancer Institute Tehran University of Medical Sciences Tehran Iran

**Keywords:** breast cancer, hormone receptor, invasive ductal carcinoma, tumor grade, ultrasound

## Abstract

**Background:**

Invasive ductal carcinoma (IDC), the most common breast cancer subtype, exhibits significant heterogeneity, limiting traditional prognostic markers. Molecular profiles improve precision, but imaging features may also reflect tumor biology.

**Aims:**

This study evaluates the predictive potential and clinical applicability of ultrasound features for determining tumor grade and molecular profiles in IDC.

**Methods and Results:**

A blinded radiologist retrospectively analyzed 109 IDC cases using the American College of Radiology (ACR) Breast Imaging Reporting and Data System (BI‐RADS) classification, evaluating ultrasound features such as lesion shape, margins, orientation, echo pattern, calcifications, vascularity, and lymph node involvement. Tumors were graded histologically (Scarff‐Bloom‐Richardson system) as low (grades 1 and 2) or high (grade 3). Immunohistochemistry determined estrogen receptor (ER), progesterone receptor (PR), human epidermal growth factor receptor 2 (HER2), and Ki‐67 status. ER and PR positivity were defined as > 10% nuclear staining, HER2 graded on a 0–3+ scale, and Ki‐67 positivity as ≥ 10% staining. Statistical analyses, including logistic and linear regression, examined correlations between ultrasound features and histological/molecular profiles. Among 109 women (mean age 48.4 ± 12.5 years), the mean tumor length and width were 21.83 ± 11.22 mm and 15.3 ± 6.97 mm, respectively. Histopathological grading revealed that grade 2 tumors were predominant (51%), while grade 1 and grade 3 tumors were observed in 25% and 24% of cases, respectively. ER and PR positivity were observed in 76.4% and 67.6% of cases, respectively. High‐grade tumors were significantly associated with ER and PR negativity (*p*‐value < 0.05). Ultrasound features associated with high‐grade tumors included larger tumor length (*p*‐value = 0.029). ER positive tumors had smaller axillary lymph nodes (*p*‐value < 0.05). Likewise, PR positive tumors exhibited smaller suspicious axillary lymph nodes compared to PR negative cases (*p*‐value = 0.004).

**Conclusion:**

Sonographic features may correlate with histological grades and hormone receptor statuses in breast IDC, suggesting that ultrasound could aid in predictive assessment.

## 1 Introduction

Breast cancer is the most prevalent type of cancer globally and represents a significant public health challenge, ranking as the fifth leading cause of cancer related mortality [1]. The World Health Organization (WHO) identifies 18 distinct histological subtypes of breast cancer, with invasive ductal carcinoma (IDC), now classified as invasive breast cancer of no special type (NST), comprising 50%–75% of all invasive cases and standing as the predominant subtype [2, 3, 4].

Established prognostic factors in breast cancer include tumor size, lymph node status, histological grade, age, ethnicity, etc., which encompass a range of clinicopathological and biological features [5]. However, the heterogeneity of breast cancer limits the precision of these indicators. Advances in molecular profiling have introduced biomarkers such as estrogen receptor (ER), progesterone receptor (PR), human epidermal growth factor receptor 2 (HER2), and Ki‐67, which enhance prognostic accuracy and guide treatment decisions [6]. These molecular subtypes exhibit distinct biological behaviors, treatment sensitivities, and growth patterns, leading to variations in imaging characteristics [7, 8, 9].

Imaging techniques, including mammography, magnetic resonance imaging (MRI), and breast ultrasound, play critical roles in breast cancer detection and management. Ultrasound, due to its accessibility, safety, and utility in lesion characterization and biopsy guidance, is a widely used diagnostic tool. Beyond its established use in detecting lesions and guiding biopsies, ultrasound has shown promise in characterizing tumor features and assessing lymph node involvement. Advances in imaging techniques and analysis raise the possibility that sonographic features may reflect underlying histological and molecular characteristics [10].

The ability to predict molecular biomarkers and histological grade characteristics from ultrasound imaging features could greatly enhance our understanding of treatment planning, prognosis, and the biological behavior of breast cancer. Emerging research suggests that ultrasound features—such as shape, margin, echogenicity, and vascularity—may correlate with histological grades and molecular markers like ER, PR, HER2, and Ki‐67 [11, 12].

IDC masses that exhibit reversed or absent diastolic flow, sharp interface boundaries, complex solid‐cystic structures, and suspicious calcifications are strongly linked to high‐grade tumors. However, these sonographic features are also common in benign lesions, complicating the differentiation between malignant and benign cases [13, 14]. Moreover, ultrasound is limited by its operator dependency, lower specificity, and difficulties in detecting breast microcalcifications [15]. These challenges highlight the need for further research into the sonographic characteristics of IDC. Despite advancements in the field, current studies remain limited in scope and fail to provide definitive insights, particularly regarding the highly prevalent and biologically diverse IDC subtypes.

Addressing this gap holds the potential to expand the role of ultrasound imaging beyond diagnosis, enabling its use as a predictive tool to assess tumor biology, guide treatment strategies, and improve prognostic accuracy. Consequently, this study aims to evaluate the associations between sonographic features of IDCs and their histological grade and molecular profiles, including ER, PR, HER2, and Ki‐67 expression. By elucidating these relationships, the research seeks to enhance the understanding of imaging biomarkers and facilitate the development of innovative, non‐invasive approaches to breast cancer management.

## 2 Materials and Methods

### 2.1 Patient Selection

We conducted a retrospective review of medical records for women with histologically confirmed IDCs who attended either our private clinic or university‐affiliated tertiary referral hospital between 2012 and 2014. Eligibility criteria included patients aged over 18 years with available ultrasound findings and histopathological reports. Patients with incomplete records or missing baseline ultrasound images were excluded from the study. The study was approved by the Research Ethics Committee of our university and adhered to the principles outlined in the Declaration of Helsinki. Given its retrospective design, the committee waived the requirement for informed consent. To ensure patient confidentiality, all data were fully anonymized in compliance with institutional regulations.

### 2.2 Ultrasound Analysis

Following standard care protocols, all patients underwent bilateral whole breast and axillary region ultrasound examinations. The imaging was performed using either an Ultrasonix Sonix OP unit (equipped with a 5–14 MHz linear probe) or an Esaote Mylab Seven unit (equipped with a 7–13 MHz linear probe), with standardized settings to minimize variability. The digitally recorded images were analyzed by an expert radiologist with 10 years of experience in breast ultrasound. All breast lesions were evaluated by a radiologist who was blinded to the patients' histopathological and molecular data, using the ultrasound classification criteria established in the 5th edition of the American College of Radiology Breast Imaging Reporting and Data System (BI‐RADS) [16]. The BI‐RADS criteria included the following features:

○ Shape: oval, round, or irregular
○ Orientation: parallel or non‐parallel
○ Margin: circumscribed or non‐circumscribed (indistinct, microlobulated, angular, spiculated)
○ Echo pattern: hypoechoic, isoechoic, hyperechoic, complex cystic, heterogeneous
○ Posterior features: no features, shadowing, enhancement, mixed pattern
○ Calcification: present or absent
○ Internal vascularity: present or absent
○ Echogenic halo: present or absent
○ Cooper's ligament changes: displacement, disruption, or no changes
○ Duct extension: present or absent
○ Number of suspicious masses in the involved breast (BI‐RADS 4 or 5, multifocal or multicentric): > 1 or 1
○ Suspicious mass in the contralateral breast: present or absent
○ Suspicious axillary lymph node: ipsilateral to the mass, bilateral, or not detected
○ Mass size: Length (mm), width (mm)
○ Maximum short‐axis diameter of suspicious axillary lymph nodes (mm)

For patients with synchronous lesions, the dominant lesion was identified by size and imaging characteristics. Ultrasound guidance ensured accurate biopsy targeting, and subsequent mastectomy with histopathological evaluation confirmed proper sampling.

### 2.3 Histological Analysis

All patients were initially diagnosed using an ultrasound‐guided core needle biopsy (CNB) before undergoing mastectomy. The resected specimens were subsequently subjected to comprehensive histopathological analysis to corroborate the CNB results. For our analysis, we relied on the mastectomy specimens. The pathologist was blinded to both the imaging and molecular findings of the cases. All breast specimens were fixed in formalin and subsequently embedded in paraffin. Tissue sections were stained with hematoxylin and eosin for histopathological examination. Tumor grading was performed using the modified Scarff‐Bloom‐Richardson system [17], categorizing tumors into grade 1 (well differentiated), grade 2 (moderately differentiated), and grade 3 (poorly differentiated). For analytical purposes, grades 1 and 2 were grouped as low‐grade tumors, while grade 3 was considered high‐grade.

### 2.4 Immunohistochemistry (IHC) Analysis

The expression status of ER, PR, HER2, and Ki‐67 was determined using IHC analysis with specific antibodies. ER and PR were classified as positive if more than 10% of nuclei showed staining. HER2 expression was graded on a scale from 0 to 3+, with grades 0 and 1 considered negative, grade 2 as borderline, and grade 3 as positive. Ki‐67 expression was quantified as the percentage of Ki‐67 positive cells among the total tumor cells counted. Ki‐67 was categorized as negative if less than 3% of cells were stained, borderline if between 3% and 10%, and positive if 10% or more were stained [18, 19]. IHC results, including ER, PR, HER2, and Ki‐67 status, were extracted from patient records. Tumors were categorized into two subtypes based on ER, PR, and HER2 status: triple‐negative breast cancer (TNBC), lacking expression of all three markers (ER, PR, HER2), and non‐TNBC, which expresses at least one of them.

### 2.5 Statistical Analysis

Statistical analyses were conducted using IBM SPSS Statistics for Windows, Version 26.0 (IBM Corp., Armonk, NY, USA). Continuous variables were reported as mean ± standard deviation (SD) and analyzed using Student's t‐test or one‐way ANOVA for group comparisons. Categorical variables were presented as frequencies and percentages, with associations assessed using chi‐square or Fisher's exact tests. In order to control for the increased risk of Type I errors due to multiple comparisons, we adjusted all *p*‐values using the Benjamini–Hochberg (BH) method. A *p*‐value of < 0.05 was considered statistically significant.

## 3 Results

### 3.1 Patient Characteristics

From an initial cohort of 620 patients, 109 women were included in the final analysis after excluding those with incomplete records or missing imaging data. The study population had a mean age of 48.4 ± 12.5 years (range: 26–80 years), and 18% of these patients were referred from a private clinic. Among the 105 patients with available menopausal status, 66 (63%) were premenopausal, and 39 (37%) were postmenopausal. Family history data were available for 106 patients: four patients had a personal history of breast cancer, eight patients had a positive family history, and 94 patients reported no family history of breast cancer. The lesions were located in the right breast in 57 patients (52.3%) and in the left breast in 52 patients (47.7%). A total of 109 dominant breast masses (one dominant mass per patient) were evaluated. Physical examination data were available for 104 patients. The dominant lesion was most commonly located in the upper outer quadrant (UOQ) in 55 patients. Other lesion locations included the upper inner quadrant (UIQ) in 12 patients, the lower outer quadrant (LOQ) in eight patients, the lower inner quadrant (LIQ) in nine patients, the retro‐areolar region in nine patients, the 12 o'clock position in eight patients, and the 6 o'clock position in three patients.

### 3.2 Ultrasound Results

The mean length and width of the breast masses were 21.83 ± 11.22 mm (range: 6–90 mm) and 15.3 ± 6.97 mm (range: 5.5–47 mm), respectively. The average diameter of the largest suspicious lymph nodes was 15.14 ± 4.5 mm.

The characteristics of the breast lesions are summarized in detail in Tables 1, 2, 3.

**TABLE 1:** Correlation between sonographic features and tumor grade in invasive ductal carcinoma of the breast.

Ultrasound features (# patients)			Tumor grade		*p* (Adjusted *p*)
			Low	High	
Mass shape (109)		Irregular (101)	91	25	0.035 (0.595)
		Oval (7)	7	0	
		Round (1)	0	1	
Mass margin (105)	Circumscribed		3	0	0.678 (0.993)
	Non‐circumscribed	Spiculated	33		
		Microlobulated	31		
		Indistinct	8		
		Angular	6		
Mass orientation (109)		Non‐parallel	70	23	0.757 (1)
		Parallel	13	3	
Mass echo pattern (108)		Hypoechoic	72	23	0.155 (0.818)
		Heterogeneous	9	1	
		Complex cyst	1	2	
Mass posterior characteristics (109)		No change	62	23	0.139 (0.818)
		Shadowing	21	3	
Mass calcification (109)		Absent	65	16	0.088 (0.802)
		Present	18	10	
Mass vascularity (105)		Not detected	64	21	0.387 (0.945)
		Hypovascular	12	5	
		Hypervascular	3	0	
Echogenic halo (108)		Absent	66	19	0.42 (1)
		Present	16	7	
Cooper's ligament changes (109)		Disruption	45	16	0.757 (1)
		No change	31	9	
		Displacement	7	1	
Duct extension (109)		Absent	77	21	0.128 (0.813)
		Present	6	5	
Suspicious mass in the involved breast (109)		=1	70	19	0.246 (0.988)
		> 1	13	7	
Suspicious mass in the contralateral breast (109)		Absent	81	25	0.56 (1)
		Present	2	1	
Suspicious axillary lymph node (108)		Absent	62	15	0.056 (0.803)
		Present, ipsilateral to mass	17	11	
		Present, both sides	3	0	

**TABLE 2:** Association of sonographic features with estrogen receptor (ER) and progesterone receptor (PR) status in invasive ductal carcinoma of the breast.

Ultrasound features			ER status			PR status		
			Positive (N)	Negative (N)	*p* (Adjusted *p*)	Positive (N)	Negative (N)	*p* (Adjusted *p*)
Mass shape		Irregular	53	15	0.210 (0.842)	46	22	0.972 (1)
		Oval	2	1		2	1	
		Round	0	1		0	0	
Mass margin	Circumscribed		1	0	0.083 (0.802)	1	0	0.326 (0.949)
	Non‐circumscribed	Spiculated	27	3		24	6	
		Microlobulated	18	8		15	11	
		Indistinct	3	3		3	2	
		Angular	5	2		4	3	
Mass orientation		Non‐parallel	46	14	1 (1)	39	20	0.739 (1)
		Parallel	9	3		9	3	
Mass echo pattern		Hypoechoic	46	16	0.157 (0.849)	40	22	0.604 (1)
		Heterogeneous	7	0		6	1	
		Complex cyst	1	1		1	0	
Mass posterior characteristics		No change	39	16	0.056 (0.803)	37	17	1.00 (1)
		Shadowing	16	1		11	6	
Mass calcification		Absent	43	12	0.527 (0.974)	38	16	0.389 (0.945)
		Present	12	5		10	7	
Mass vascularity		Not detected	44	15	0.788 (1)	38	20	0.267 (0.949)
		Hypovascular	9	2		9	2	
		Hypervascular	1	0		0	1	
Echogenic halo		Absent	43	15	0.495 (0.974)	39	18	0.759 (1)
		Present	12	2		9	5	
Cooper's ligament changes		Disruption	29	9	0.921 (1)	24	13	0.865 (1)
		No change	24	7		22	9	
		Displacement	2	1		2	1	
Duct extension		Absent	50	16	1.00 (1)	46	19	0.082 (0.849)
		Present	5	1		2	4	
Suspicious mass in the involved breast		=1	46	10	0.046 (0.803)	40	16	0.221 (0.949)
		> 1	9	8		8	7	
Suspicious mass in the contralateral breast		Absent	54	17	0.576 (1)	47	23	1.0 (1)
		Present	1	0		1	0	
Suspicious axillary lymph node		Absent	41	9	0.108 (0.849)	37	13	0.059 (0.803)
		Present, ipsilateral to mass	11	8		8	10	
		Present, both sides	2	0		2	0	

Abbreviations: ER, estrogen receptor; N, number; PR, progesterone receptor.

**TABLE 3:** Associations between sonographic features and HER2, Ki‐67, and triple‐negative breast cancer (TNBC) status in invasive ductal carcinoma of breast.

Ultrasound features			HER2 status				Ki‐67 status				TNBC status		
			Positive	Borderline	Negative	*p* (Adjusted *p*)	Positive	Borderline	Negative	*p* (Adjusted *p*)	Positive	Negative	*p* (Adjusted *p*)
Mass shape		Irregular	24	6	36	0.267 (0.949)	13	1	2	0.678 (0.993)	7	61	1 (1)
		Oval	2	0	0		2	0	0		0	3	
		Round	0	0	1		0	0	0		0	0	
Mass margin	Circumscribed		1	0	0	0.567 (1)	0	0	0	0.842 (1)	0	1	0.271 (0.974)
	Non‐circumscribed	Spiculated	11	2	15		7	1	1		1	29	
		Microlobulated	8	2	15		4	0	1		5	21	
		Indistinct	1	2	3		1	0	0		0	5	
		Angular	3	0	4		3	0	0		1	6	
Mass orientation		Non‐parallel	23	6	29	0.191 (0.949)	12	1	2	0.547 (1)	5	54	0.337 (0.974)
		Parallel	3	0	8		3	0	0		2	10	
Mass echo pattern		Hypoechoic	24	4	32	0.601 (1)	11	1	2	1.00 (1)	7	55	1.00 (1)
		Heterogeneous	2	1	3		4	0	0		0	7	
		Complex cyst	0	0	2		0	0	0		0	1	
Mass posterior characteristics		No change	17	4	32	0.093 (0.849)	15	1	2	—	7	47	0.185 (0.974)
		Shadowing	9	2	5		0	0	0		0	17	
Mass calcification		Absent	20	4	29	0.759 (1)	13	1	2	1.00 (1)	4	50	0.346 (1)
		Present	6	2	8		2	0	0		3	14	
Mass vascularity		Not detected	23	8	37	0.460 (1)	6	8	1	0.378 (0.974)	6	61	0.691 (1)
		Hypovascular	5	0	9		4	2	2		2	12	
		Hypervascular	1	0	1		1	0	0		0	2	
Echogenic halo		Absent	20	3	33	0.703 (1)	12	1	2	0.547 (1)	0	14	0.331 (0.974)
		Present	6	3	4		3	0	0		7	50	
Cooper's ligament changes		Disruption	13	4	20	0.790 (1)	10	0	1	0.168 (0.949)	2	35	0.28 (1)
		No change	11	2	16		5	1	0		4	27	
		Displacement	2	0	1		0	0	1		1	2	
Duct extension		Absent	22	6	35	0.259 (0.949)	15	1	2	—	7	58	1.00 (1)
		Present	4	0	2		0	0	0		0	6	
Suspicious mass in the involved breast		=1	22	4	27	0.437 (1)	14	1	1	0.314 (0.974)	5	51	0.634 (1)
		> 1	4	2	10		1	0	1		2	13	
Suspicious mass in the contralateral breast		Absent	26	6	36	0.533 (1)	15	1	2	1 (1)	63	7	1.00 (1)
		Present	0	0	1		0	0	0		1	0	
Suspicious axillary lymph node		Absent	16	5	26	0.5 (1)	9	1	2	0.67 (1)	5	45	1.00 (1)
		Present, ipsilateral to mass	10	1	8		6	0	0		2	16	
		Present, both sides	0	0	2		0	0	0		0	2	

Abbreviations: HER2, human epidermal growth factor 2; N, number; TNBC, triple‐negative breast cancer.

**FIGURE 1:**
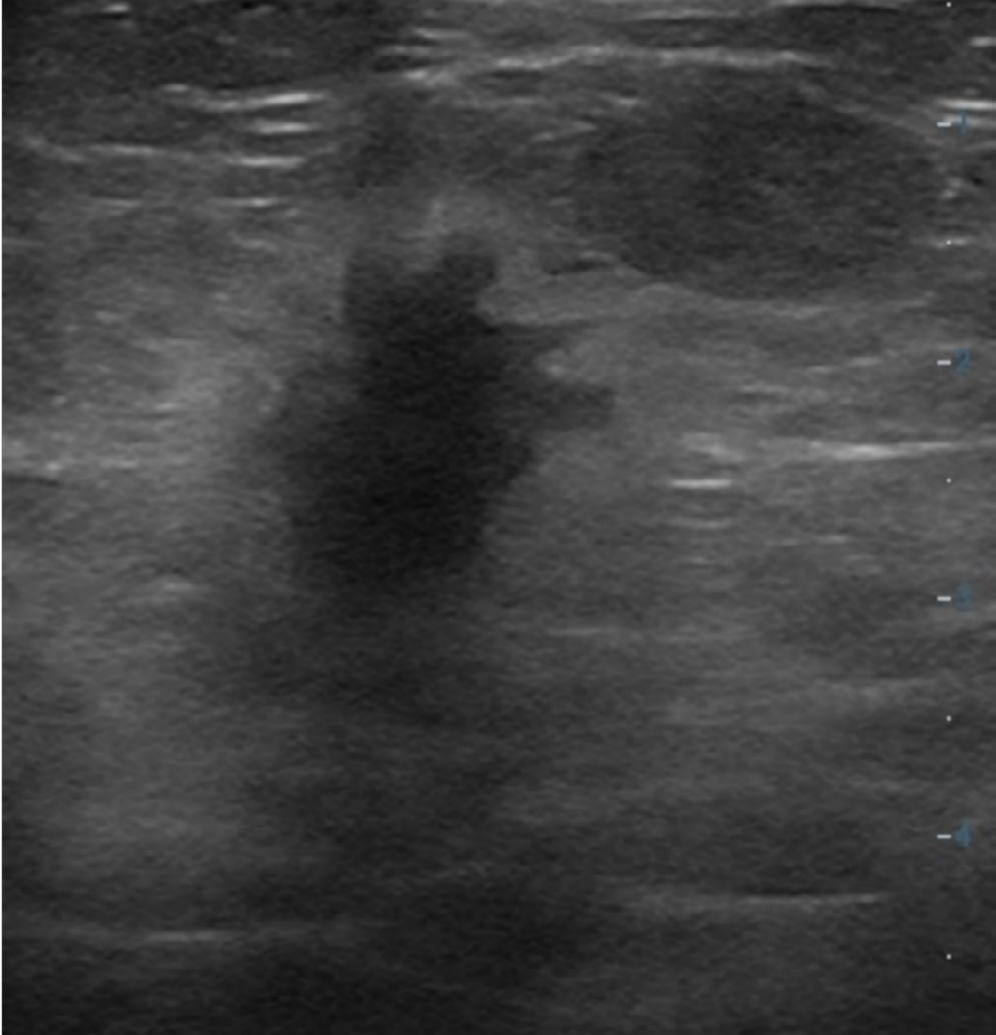
Grayscale ultrasound image of a 57‐year‐old woman with a left breast mass. The lesion exhibits a hypoechoic echo pattern, non‐parallel orientation, irregular shape, spiculated margins, posterior acoustic shadowing, and a surrounding echogenic halo. Histopathological and immunohistochemical analysis confirmed a grade 1 invasive ductal carcinoma (IDC) that is ER positive, PR positive, HER2 negative, with a Ki‐67 index of 6%.

**FIGURE 2:**
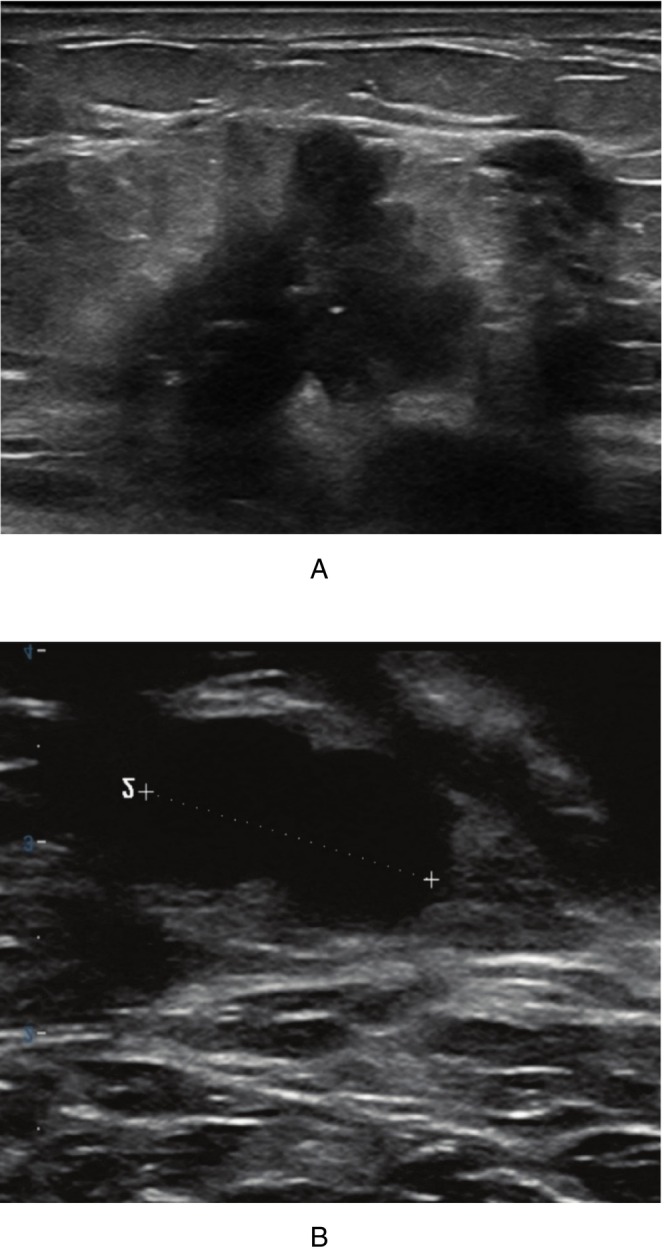
Grayscale ultrasound images of a 65‐year‐old woman with a right breast mass. (A) Shows a hypoechoic mass with parallel orientation, irregular shape, microlobulated margins, posterior acoustic shadowing, and a surrounding echogenic halo. (B) Illustrates a suspicious hypoechoic ipsilateral axillary lymph node without an echogenic hilum Histopathological and immunohistochemical analysis confirmed a grade 3 invasive ductal carcinoma (IDC) that is ER positive, PR negative, HER2 negative, with a Ki‐67 index of 15%. The axillary lymph node was involved by IDC.

**FIGURE 3:**
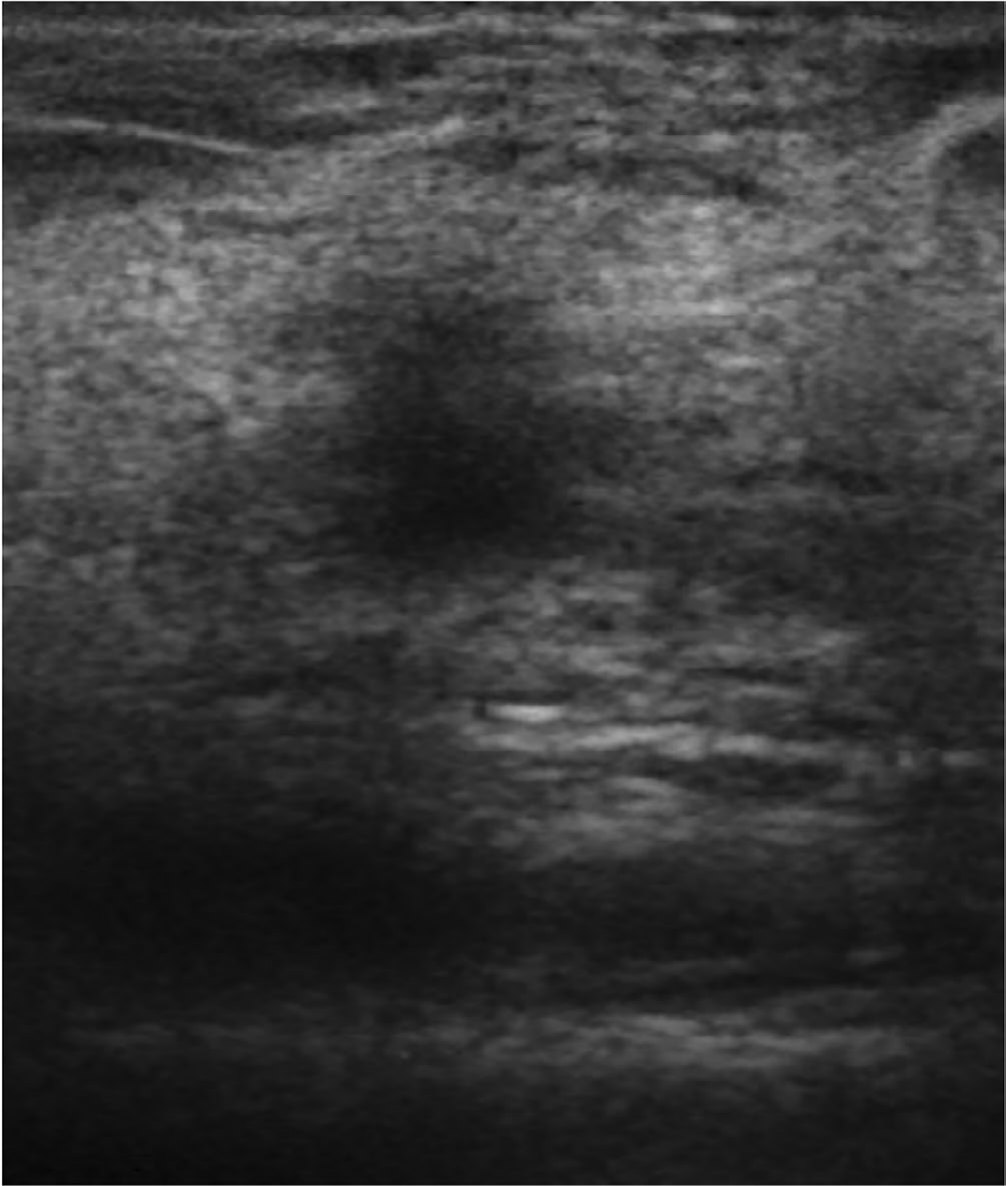
A grayscale ultrasound image of a 43‐year‐old female presenting with a left breast mass. The image shows a hypoechoic mass characterized by a non‐parallel orientation, irregular shape, indistinct margins, and a surrounding echogenic halo. Histopathological and immunohistochemical analysis confirmed the mass to be a grade 1 invasive ductal carcinoma (IDC) that was ER positive, PR positive, HER2 positive, with a Ki‐67 proliferation index of 4%.

Figures 1, 2, 3 illustrate ultrasound findings in three patients with breast masses, showcasing various sonographic features such as hypoechoic lesions, irregular shapes, non‐parallel orientation, spiculated or indistinct margins, posterior acoustic shadowing, and echogenic halos. These sonographic patterns were frequently observed in cases with specific histopathological and molecular features, as illustrated in Figures 1, 2, 3. This highlights the diagnostic utility of ultrasound imaging in evaluating breast lesions.

### 3.3 Histopathology and Immunohistochemistry Results

Due to missing data, ER status was available for 72 cases, and PR for 71 cases. Among the evaluated biomarkers, ER expression was the most prevalent, detected in 55 of 72 tumors (76.4%), followed by PR expression, which was identified in 48 of 71 tumors (67.6%). The predominant pathological grade was grade 2, observed in 56 patients (51.4%), while grade 1 was noted in 27 patients (24.8%) and grade 3 in 26 patients (23.9%). Negative ER, negative PR, and TNBC statuses were more commonly observed in high‐grade IDCs compared to low‐grade tumors. The analysis demonstrated that histologic grading was significantly associated with molecular markers, including ER and PR (Adjusted *p*‐value = 0.0405) (Table 4).

**TABLE 4:** Distribution of biological markers, tumor grades, and their correlation in patients with invasive ductal carcinoma.

Biological markers (#lesions)		Tumor grade (*N* = 109)		*p* (Adjusted *p*)
		Low (*N* = 83)	High (*N* = 26)	
ER (*N* = 72)	Positive (*N* = 55)	49	6	< 0.001* (0.0405*)
	Negative (*N* = 17)	6	11	
PR (*N* = 71)	Positive (*N* = 48)	44	4	< 0.001* (0.0405*)
	Negative (*N* = 23)	11	12	
HER2 (*N* = 69)	Positive (*N* = 26)	17	9	0.351 (0.812)
	Intermediate (*N* = 6)	4	2	
	Negative (*N* = 37)	31	6	
Ki‐67 (*N* = 18)	Positive (*N* = 15)	10	5	0.337 (0.812)
	Intermediate (*N* = 1)	1	0	
	Negative (*N* = 2)	2	0	
TNBC (*N* = 71)	Positive (*N* = 7)	4	3	0.041* (0.597)
	Negative (*N* = 64)	12	52	

Abbreviations: ER, estrogen receptor; HER2, human epidermal growth factor 2; N, number; PR, progesterone receptor; TNBC, triple‐negative breast cancer.

* Statistically significant.

### 3.4 Correlation Between Ultrasound Findings and Tumor Grade

Table 1 summarizes the correlation between ultrasound findings and tumor grade.

The analysis revealed that the mean tumor length was 20.37 ± 8.57 mm in low‐grade tumors and 26.28 ± 16.7 mm in high‐grade tumors, with a statistically significant difference (*p*‐value = 0.029). The mean tumor width was 14.56 ± 6.05 mm for low‐grade tumors and 17.79 ± 9.23 mm for high‐grade tumors, though this difference was not statistically significant (*p*‐value = 0.084). The mean diameter of the largest suspicious axillary lymph node was 13.5 ± 4.4 mm in low‐grade tumors and 18 ± 3.4 mm in high‐grade tumors. Although this difference did not reach statistical significance (*p*‐value = 0.075), the trend suggests a potential association.

### 3.5 Correlation Between Ultrasound Features and Biological Markers

#### 3.5.1 Correlation Between Ultrasound Features and ER/PR Expression

Table 2 delineates the correlation between sonographic features and ER/PR status in IDC. The analysis revealed that irregular shape, spiculated margins, non‐parallel orientation, hypoechoic pattern, Cooper's ligament disruption, solitary mass in the affected breast, and smaller diameters of maximum lymph nodes were more frequently observed in ER‐positive IDC cases, which were 11.8 ± 2.38 vs. 20.3 ± 1.52 mm in ER‐negative cases. The mean tumor length and width were 19.97 ± 8.01 and 14.65 ± 6.28 mm, respectively, in ER‐positive cases, compared to 22.8 ± 11.44 and 16.33 ± 7.61 mm in ER‐negative cases. Although differences in lesion size and shape were observed between ER‐positive and ER‐negative tumors, these did not reach statistical significance.

The mean diameter of the largest suspicious axillary lymph node was significantly smaller in PR positive cases (11 ± 1.82 mm) compared to PR negative cases (19 ± 2.94 mm) (*p*‐value = 0.004).

#### 3.5.2 Correlation Between Ultrasound Features and HER2, Ki‐67, and TNBC Status

Table 3 represents the associations between ultrasound findings and HER2, Ki‐67, and TNBC status. In this study, no sonographic features demonstrated a statistically significant association with HER2 expression. However, differences in tumor dimensions based on HER2 status were noted. Specifically, the mean tumor length was 21.07 ± 8.99 mm in HER2 positive cases, 14.25 ± 7.36 mm in borderline cases, and 21.04 ± 9.57 mm in HER2 negative cases (*p*‐value = 0.163). The mean tumor width was 15.88 ± 5.54, 9.12 ± 2.52, and 15.78 ± 7.16 mm for HER2 positive, borderline, and negative cases, respectively (*p*‐value = 0.140). Further evaluation of axillary involvement showed that the mean diameter of the largest suspicious axillary lymph node was 15.2 ± 4.32 mm in HER2 positive cases and 14.66 ± 6.65 mm in HER2 negative cases, without significant differences (*p*‐value = 0.893).

No ultrasound findings demonstrated a significant correlation with TNBC status in IDC cases. In TNBC cases, the mean tumor length was larger at 21.41 ± 7.08 mm, compared to 19.86 ± 7.69 mm in non‐TNBC patients, though this difference was not statistically significant (*p*‐value = 0.640). The mean tumor width in TNBC cases was 14.5 ± 3.61 mm, compared to 14.55 ± 6.15 mm in non‐TNBC cases (*p*‐value = 0.982). The mean diameter of the largest suspicious axillary lymph node was larger in TNBC cases (22 mm) compared to non‐TNBC cases (14 mm) (*p*‐value = 0.128).

In cases assessed for Ki‐67 expression, the mean tumor length was 24.25 ± 9.08 mm in positive cases and 17.5 ± 3.5 mm in negative cases (*p*‐value = 0.173). Ki‐67 intermediate expression was observed in only one case (length: 39 mm), limiting interpretation for this group. The mean tumor width was 19.04 ± 7.22 mm in positive cases, 34 mm in one intermediate case, and 12.5 ± 2.12 mm in negative cases (*p*‐value = 0.084).

## 4 Discussion

In this study, we employed ultrasound imaging, a broadly accessible technique, to evaluate IDCs. Ultrasound plays a crucial role in distinguishing between malignant and benign breast lesions. Relative to mammography, ultrasound demonstrates greater sensitivity and provides a more practical combination of accessibility and diagnostic effectiveness [20]. Our findings indicate that high‐grade IDCs are generally characterized by increased tumor length and distinct mass shapes when compared to low‐grade lesions. This observation aligns with the study conducted by Gupta et al., which demonstrated a significant association between mass shape observed on ultrasound and the histological grade of IDCs [14].

Irshad et al. demonstrated that posterior acoustic shadowing was significantly associated with low‐grade tumors, with an odds ratio exceeding 13 [12]. In our cohort, posterior acoustic shadowing was identified in 25% of low‐grade tumors and 11.5% of high‐grade tumors; however, this difference was not statistically significant (*p* = 0.818). This variance underscores the biological heterogeneity of IDC and suggests that ultrasound may reflect a wider range of tumor characteristics than previously appreciated [21]. This discrepancy highlights the potential variability in sonographic presentations of IDC grades and suggests that certain ultrasound features like posterior shadowing may not be consistent indicators of tumor grade across different cohorts.

Studies have shown that high‐grade IDCs commonly exhibit characteristic ultrasound features, including increased tumor size, pronounced hypoechogenicity, absent or reversed diastolic flow, sharply defined tumor‐tissue interfaces, and calcifications located within or surrounding the lesion [14, 22]. However, some studies have reported variability in these characteristics. For example, Watermann et al. found no significant correlation between tumor grade and ultrasound features [23] while Lamb et al. noted that high‐grade tumors may paradoxically present with a “benign” appearance on ultrasound, characterized by nonspecific sonographic features [24]. These findings align with our observations, suggesting that sonographic features alone may not reliably predict the histological grade of IDCs.

ER and PR statuses are crucial prognostic and therapeutic markers in breast cancer. Although not statistically significant, ER positive lesions in our cohort more frequently presented as solitary masses exhibiting spiculated or microlobulated margins, reflecting trends noted in prior studies. Similarly, PR positive lesions were associated with smaller axillary lymph nodes. These features are consistent with previous studies, which have reported that ER positive lesions often exhibit non‐circumscribed margins and posterior acoustic shadowing [12, 25, 26]. PR status has been associated with distinct sonographic features in earlier studies. Zhang et al. reported that PR positive lesions often present as smaller masses with lobulated, angular, or spiculated margins and typically lack calcifications [25]. Other research has noted significant differences in posterior shadowing and echo patterns between PR positive and PR negative lesions [12, 26, 27]. However, our study did not identify significant associations between PR status and these features. The discrepancies between our findings and previous reports may be attributed to differences in sample size, imaging techniques, or patient populations. This highlights the need for further investigation to determine whether PR status consistently influences sonographic appearance or if these associations are influenced by external factors such as imaging variability or tumor heterogeneity.

Similarly, HER2 status, which is associated with aggressive tumor behavior, has been linked to specific imaging features in breast ultrasound. HER2 overexpression is known to correlate with adverse prognostic factors, including larger tumor size, higher grade, and increased metastatic potential. Prior research has suggested that HER2 positive lesions frequently exhibit architectural distortion, indistinct or spiculated margins, calcifications, and posterior enhancement [28]. For instance, Shin et al. observed a significant association between HER2 positivity and the presence of calcifications. Wojcinski et al. also noted greater architectural distortion in HER2 positive cases [29, 30]. Furthermore, there is evidence to suggest that features like indistinct or spiculated margins and posterior enhancement are more prevalent in HER2 positive invasive breast cancer [31, 32, 33]. Despite these established findings, our study did not find any significant correlations between HER2 status and sonographic features.

TNBC, an aggressive subtype of IDC, is another molecular marker of interest. Previous studies have consistently shown that TNBC lesions often present with distinctive sonographic features, including circumscribed, indistinct, or microlobulated margins, hypoechoic appearance, and round, oval, or lobulated shapes with an abrupt interface [29, 34, 35, 36, 37]. In contrast, non‐TNBCs are more likely to exhibit irregular shapes and spiculated margins [38]. Early detection is particularly critical in TNBC due to its high responsiveness to neoadjuvant chemotherapy. Despite the strong associations reported in the literature, our study did not observe significant correlations between sonographic features and TNBC status. This may be due to the limited number of TNBC cases in our cohort, which could have reduced the statistical power needed to detect meaningful differences.

Ki‐67, a well‐established proliferation marker, plays a dual role as both a prognostic factor and a predictor of therapeutic response. Higher Ki‐67 expression has been associated with increased sensitivity to neoadjuvant chemotherapy, making it a key biomarker in clinical decision making [39]. While thresholds such as ≥ 14% and ≥ 20% are widely recognized in breast cancer, particularly following the St. Gallen International Expert Consensus, our study employed a more conservative categorization (< 3% as negative, 3%–10% as borderline, and ≥ 10% as positive). This categorization was adopted to investigate the potential prognostic implications of lower Ki‐67 expression levels in our patient population, acknowledging the variability and lack of consensus on standardized cutoff values in breast cancer studies [40]. Some studies, such as Li et al., have reported that Ki‐67‐positive lesions frequently demonstrate sonographic features like posterior enhancement and spiculated margins [41]. However, our study did not find significant associations between Ki‐67 expression and sonographic features. This discrepancy could be attributed to the retrospective nature of our study and the incomplete availability of Ki‐67 data across all patients, which might have introduced bias or limited our ability to draw definitive conclusions.

To our knowledge, this is among the few studies to comprehensively evaluate the correlation between BI‐RADS ultrasound features and multiple molecular subtypes—including Ki‐67 and TNBC status—in a well‐defined IDC cohort. This study offers several key strengths. First, it focuses on IDCs, the most common type of breast cancer, ensuring the findings are relevant to a large patient population. Additionally, it explores the relationship between sonographic features, histological grades, and molecular markers, utilizing ultrasound—a widely available, non‐invasive imaging technique commonly used in clinical practice. These detailed associations illustrate the potential of breast ultrasound to characterize tumor biology, offering prognostic insights that go beyond conventional metrics like size and nodal involvement. By integrating sonographic findings with histopathological and molecular data, our study underscores the role of ultrasound in tailoring breast cancer management. The study's rigorous design, which included a blinded review of sonographic images alongside pathological and histological data, minimizes observer bias and ensures the reliability of the results.

Our study explored the potential of ultrasound in predicting tumor grade and molecular markers in breast cancer; however, several expected associations, such as HER2 and TNBC status, did not reach statistical significance. This may be attributed to tumor heterogeneity, the macroscopic nature of ultrasound imaging, and confounding biological factors that influence imaging characteristics. While ultrasound provides valuable morphological and vascular information, it has inherent limitations in distinguishing molecular subtypes, as no imaging modality alone can reliably replace histopathological and immunohistochemical analysis. Advanced imaging techniques such as MRI, elastography, and PET/CT have demonstrated potential in correlating with tumor biology, particularly in differentiating aggressive subtypes like TNBC and HER2 positive tumors based on perfusion, metabolic activity, and tissue stiffness. However, these imaging features are not yet definitive biomarkers for molecular classification, reinforcing the need for a multimodal diagnostic approach that integrates imaging with pathological assessment.

Our study is also limited by its retrospective design and reliance on previously acquired images for analysis. To minimize bias, an experienced investigator, blinded to clinical outcomes, thoroughly reviewed all examinations, and standardized imaging protocols were employed to enhance reliability. Despite these efforts, a notable limitation is the incomplete availability of molecular parameters for all patients, which may affect the robustness and generalizability of our findings. Additionally, the study's small sample size and potential imaging variability may have influenced the results, emphasizing the need for larger, multicenter studies with standardized imaging protocols to further validate these associations.

## 5 Conclusion

This study identified a potential correlation between ultrasound features and both the histological grade and biological markers in IDCs. These findings suggest that breast ultrasound may have a role in prognostic assessments and guiding treatment strategies. However, further studies on larger and more comprehensive datasets are needed to confirm these results.

## Author Contributions

**Golnaz Moradi:** conceptualization, methodology, investigation, resources, writing – original draft, writing – review and editing, visualization, supervision, project administration. **Nasrin Ahmadinejad:** conceptualization, methodology, investigation, resources, writing – original draft, writing – review and editing, visualization, supervision, project administration. **Diana Zarei:** methodology, formal analysis, resources, writing – original draft, writing – review and editing, visualization. **Nahid Sadighi:** methodology, investigation, resources, writing – original draft, writing – review and editing, visualization.

## Ethics Statement

This study was approved by the ethics committee of Tehran University of Medical Sciences (TUMS).

## Consent

Written informed consent was waived by the Ethics Research Committee of Tehran University of Medical Sciences (TUMS) due to the study's retrospective design.

## Conflicts of Interest

The authors declare no conflicts of interest.

## Data Availability

The data that support the findings of this study are available from the corresponding author upon reasonable request.
